# Immunotherapy in Bellini Duct Carcinoma: A Systematic Review

**DOI:** 10.32604/or.2026.081674

**Published:** 2026-09-14

**Authors:** Antonio David Lázaro-Sánchez, Javier David Benítez-Fuentes, Sofía Wikström-Fernández, María Nevado-Rodríguez, Pablo Conesa-Zamora, Ginés Luengo-Gil, Alejandra Ivars-Rubio, Marta Zafra-Poves, Edgardo D. Carosella, Belén Fernández-Molina, Andrés Nieto-Olivares, María José Sánchez de las Matas Garre, Ana Belén Arroyo

**Affiliations:** 1 Department of Medical Oncology, University Clinical Hospital Virgen de la Arrixaca, Murcia, Spain; 2 Group of Molecular Pathology and Pharmacogenetics, Biomedical Research Institute from Murcia (IMIB), Laboratory Medicine and Pathology Department, Santa Lucía General University Hospital, Cartagena, Spain; 3 Department of Medical Oncology, Elche General University Hospital, Alicante, Spain; 4 Department of Medical Oncology, Santa Lucia General University Hospital, Cartagena, Spain; 5 Department of Medical Oncology, Morales Meseguer General University Hospital, Murcia, Spain; 6 Health Sciences Faculty, Universidad Católica de Murcia (UCAM), Guadalupe, Spain; 7 CEA, DRF-Institut de Biologie François Jacob, Service de Recherches en Hémato-Immunologie, Hôpital Saint-Louis, Paris, France; 8 Family and Community Medicine, Lorenzo Guirao Hospital, Murcia, Spain; 9 Department of Pathological Anatomy, Morales Meseguer General University Hospital, Murcia, Spain; 10 Department of Pathological Anatomy, Santa Lucía General University Hospital, Cartagena, Spain

**Keywords:** Collecting duct carcinoma, Bellini duct carcinoma, immunotherapy, PD-1/PD-L1 blockade, systematic review

## Abstract

**Background:** Collecting duct carcinoma (CDC; Bellini duct carcinoma) is a rare, aggressive renal cancer with no established standard of care, and evidence for immune checkpoint inhibitor (ICI)-based therapy in CDC remains emerging and fragmented. We aimed to systematically synthesise efficacy and safety data on immunotherapy in adult patients with CDC. **Methods:** PubMed and Web of Science were searched from inception to 29 March 2025, with targeted post-search monitoring of key journals and ClinicalTrials.gov updated on 11 April 2026. Prospective interventional studies, observational cohorts/registries, and case series/reports were eligible. Screening, extraction and risk-of-bias appraisal (Joanna Briggs Institute tools) were performed independently in duplicate; results were narratively synthesised. **Results:** Overall, ICIs—particularly combinations—showed clinically meaningful activity in a subset of CDC patients. Twenty-four studies met criteria (2 prospective trials, 8 observational cohorts, 14 case-level publications/16 patients). The SUNNIFORECAST randomised phase II trial reported a CDC-specific ORR of 40% with ipilimumab + nivolumab versus 20% with standard of care (n = 9 CDC). Observational evidence yielded ORR ~10–44% and mOS ~13–42 months depending on regimen, with the highest activity seen with ICI plus TKI combinations. Durable complete responses (≥3–6 years) were documented at case level with dual checkpoint blockade and anti-PD-1 monotherapy. Safety was generally manageable, though serious events occurred sporadically (immune-mediated hepatitis; one fatal pneumonia). **Conclusions:** ICIs—particularly combination strategies—demonstrate clinically meaningful activity in selected patients with CDC, but evidence is limited by small samples, heterogeneity and high risk of bias. Prospective, biomarker-informed CDC-dedicated trials and coordinated registries are needed.

## 1 Introduction

Collecting duct carcinoma (CDC), also called Bellini duct carcinoma, is a rare and aggressive variant of renal cell carcinoma, accounting for <1% of kidney tumors [1]. It typically presents at an advanced stage—40–50% of patients have metastatic disease at diagnosis—and prognosis is poor, with median survival in metastatic cases often <1 year [1]. Standard therapies for clear-cell RCC (e.g., VEGF-targeted agents) have shown limited efficacy in CDC; historically, the best outcomes have come from platinum-based chemotherapy. In pooled analyses, gemcitabine–platinum has yielded an objective response rate of roughly 26% [2]. Earlier immunotherapy approaches (interleukin-2 or interferon) were largely ineffective; a prior systematic review reported no responses among 49 patients treated before the immune-checkpoint inhibitor (ICI) era [2].

The rationale for testing immune checkpoint blockade in CDC has strengthened. Molecular and microenvironmental studies indicate an immune-infiltrated phenotype with frequent expression of PD-1/PD-L1 pathway components [3]. Transcriptomic profiling further suggests immunogenicity, supporting evaluation of checkpoint inhibition [3,4].

Clinically, immune checkpoint inhibitors (anti-PD-1/PD-L1 and anti-CTLA-4) have transformed advanced clear-cell RCC, and early signals of activity have emerged in non-clear-cell subtypes, including CDC [5]. Case reports and small series describe meaningful responses to ICIs—for example, partial responses with nivolumab monotherapy in several reports [1,5]. Retrospective analyses and early-phase studies also support activity: in a multicentre retrospective analysis of nivolumab in patients with metastatic non-clear-cell RCC, approximately 25% of CDC patients achieved an objective response [6]; additional small cohorts have reported objective responses with checkpoint inhibitors [7,8].

Despite these observations, the evidence remains fragmented. The rarity of CDC means that few studies have specifically evaluated immunotherapy in this subtype [1,9]. Accordingly, we undertook a systematic review to consolidate efficacy and safety data for immunotherapy in Bellini duct carcinoma, with the specific aim of evaluating the efficacy and safety of immunotherapy in adult patients with CDC and of identifying candidate biomarkers and combination strategies relevant to clinical decision-making.

## 2 Materials and Methods

This systematic review was designed and conducted in accordance with the Preferred Reporting Items for Systematic Reviews and Meta-Analyses (PRISMA) 2020 guidelines (Supplementary Material S1) [9]. The review protocol was established a priori and registered in the PROSPERO database (Registration ID: CRD420251022132) before commencing the study selection.

### 2.1 Eligibility Criteria

We defined inclusion and exclusion criteria as follows:

Inclusion Criteria

• Patients older than 18 years-old with histologically confirmed CDC, also known as Bellini duct carcinoma, at any disease stage.
• Any type of immunotherapy, including immune checkpoint inhibitors (anti–PD-1, anti–PD-L1, anti–CTLA-4), cytokine therapies (e.g., interleukins, interferons), cellular therapies (e.g., adoptive T-cell therapies), cancer vaccines, or combinations involving these approaches.
• Any comparator is acceptable but not required. We will include both comparative studies (e.g., trials comparing immunotherapy to another treatment) and single-arm studies/series (no specific comparator). Standard care treatments (such as chemotherapy, targeted therapy, or surgery) in comparison groups will be noted when applicable, but the absence of a comparator will not exclude a study.
• Studies must report at least one clinical outcome of interest related to treatment efficacy or safety. Efficacy outcomes of interest include tumor response rates, progression-free survival, overall survival, or disease control rate. Safety outcomes include treatment-related adverse events or toxicities. For studies enrolling mixed non–clear-cell RCC histologies, eligibility required either (i) reporting of at least one outcome (efficacy or safety) specifically for the CDC subgroup, or (ii) in the absence of CDC-disaggregated outcomes, inclusion was restricted to prospective interventional trials for which histologically confirmed CDC patients contributed to the overall reported efficacy cohort. Under rule (ii), such studies were classified as providing contextual prospective evidence rather than CDC-specific efficacy estimates, and their CDC-level outcomes were reported as not available rather than imputed.
• We will include all relevant study types that provide clinical data on immunotherapy in this cancer. Eligible designs include randomized controlled trials, non-randomized trials, single-arm phase II studies, cohort studies, retrospective analyses, case series and case reports.
• Only studies published in English will be included.

Exclusion Criteria

• Studies involving patients younger than 18 years old, or those studies focusing exclusively on other subtypes of RCC without separately reported data specifically for collecting duct carcinoma.
• Studies evaluating treatments not classified as immunotherapy, including studies focusing exclusively on chemotherapy, targeted therapies (e.g., VEGF inhibitors, mTOR inhibitors), surgery, radiation therapy, or supportive care without immunotherapy.
• Studies not reporting clinical outcomes related to treatment efficacy or safety. This includes publications that provide exclusively molecular, pathological, or imaging data without clinical patient outcomes, or studies where the outcomes of interest are not clearly reported.
• Narrative or expert reviews, systematic reviews, meta-analyses, editorials, letters to the editor, conference abstracts lacking detailed clinical data, commentaries, and opinion pieces. Additionally, preclinical studies (*in vitro* studies or animal models) or purely laboratory-based investigations without patient-related clinical outcomes are excluded.
• Publications reporting duplicate or overlapping patient cohorts or clinical data. When duplication occurs, only the most recent or the most complete publication with comprehensive patient outcomes will be included.
• Studies published exclusively in non-English languages, studies without accessible English abstracts or summaries, and studies with insufficient data in their English abstract to accurately assess the relevant outcomes or inclusion criteria.

### 2.2 Search Strategy

We performed a comprehensive search of two electronic databases: PubMed and Web of Science (PubMed: https://pubmed.ncbi.nlm.nih.gov/; Web of Science: https://www.webofscience.com/). Given that collecting duct carcinoma is an ultra-rare disease with literature concentrated in a small number of oncology journals, the overlap in CDC-specific indexing between PubMed and Embase is expected to be substantial; Embase was therefore not formally searched as a systematic database for this review, and this is acknowledged as a limitation in the Discussion. To identify relevant studies published between the original search date and manuscript submission, we performed targeted monitoring of key genitourinary-oncology journals (Annals of Oncology, European Urology, Journal of Clinical Oncology, Clinical Genitourinary Cancer, Journal for ImmunoTherapy of Cancer) and of relevant ClinicalTrials.gov records; studies identified through this targeted post-search monitoring were incorporated via the PRISMA 2020 “other methods” pathway rather than via the formal database search, and are tabulated separately in the PRISMA flow diagram. Grey literature (conference abstracts from ASCO, ESMO and ASCO-GU) was searched opportunistically but not systematically; this is also acknowledged as a limitation. Language restriction was set to English; this introduces a potential language bias given the substantial number of Japanese- and Chinese-language case reports on CDC, and is also acknowledged as a limitation in the Discussion. The PubMed and Web of Science search was performed on 11 April 2026 (updated from an initial search on 29 March 2025). For the PubMed search, we used the following query:

(“collecting duct carcinoma” OR “Bellini duct carcinoma” OR “renal collecting duct carcinoma”) AND (“immunotherapy” OR “immune checkpoint inhibitor” OR “PD-1” OR “PD-L1” OR “CTLA-4” OR “immune therapy” OR “checkpoint blockade”)

The PubMed strategy was then adapted to the syntax of the Web of Science database. No date restrictions were placed on the search; articles were included from database inception until the search date.

### 2.3 Study Selection

The study selection was conducted independently by four reviewers (ADLS, JDBF, SW, and MNR: Antonio David Lazaro Sanchez, Javier David Benitez Fuentes, Sofía Wikström, and María Nevado Rodríguez) through a two-step screening process. Initially, titles and abstracts retrieved from the database searches were reviewed to identify potentially relevant studies according to the eligibility criteria. Subsequently, the full texts of potentially relevant articles were independently reviewed by the same reviewers to finalize study inclusion. Full-text assessment was performed independently and in duplicate by pairs of reviewers, who were blinded to each other’s decisions during the initial appraisal, in order to minimise selection bias. Any disagreements during the screening or full-text assessment phases were resolved by consensus through discussions involving two additional reviewers (BFM and ABA). Reasons for exclusion at the full-text stage were documented, and the entire selection process was reported using a PRISMA flow diagram. Additional records were identified through two complementary non-database pathways, both summarised in the PRISMA flow diagram under the “other methods” identification branch. First, backward citation searching (reference-list screening of included studies and key narrative reviews in the field) yielded 5 additional records. Second, targeted post-search monitoring of high-impact genitourinary-oncology journals and of relevant ClinicalTrials.gov records identified 2 further key publications that appeared between the original search date and manuscript submission: the SUNNIFORECAST randomised trial with a CDC subgroup and the Zeng et al., 2025 observational series. All 7 records thus identified were assessed at full-text level against the same eligibility criteria applied to database-identified records. The full-text assessment of these non-database records was carried out independently and in duplicate by the same four reviewers (ADLS, JDBF, SW, MNR) following the identical screening procedure used for database-identified records, with discrepancies resolved by consensus with the two senior reviewers (BFM and ABA: Belén Fernández Molina, and Ana Belén Arroyo).

### 2.4 Data Extraction

Data extraction from included studies was independently carried out by four reviewers (ADLS, JDBF, SW, and MNR: Antonio David Lazaro Sanchez, Javier David Benitez Fuentes, Sofía Wikström, and María Nevado Rodríguez) using a standardized data extraction form tailored for this review. Information extracted included publication details (author, year, country), study design, number of patients and patient characteristics (age, sex, disease stage), details of the immunotherapy regimen (agents, dosage, duration), comparators when applicable, and outcomes (tumor response rates, progression-free survival, overall survival, and adverse events). Missing or incomplete data were handled as follows: when an outcome of interest was not reported in the primary publication, the corresponding author of the source study was contacted by email to request clarification or additional information; when no response was obtained or the data could not be retrieved, the variable was recorded as “not reported” (NR) in the data extraction tables and was not imputed. No assumptions were made about missing values, and analyses were conducted on the basis of the data actually available for each study, with the corresponding limitations explicitly acknowledged in the synthesis and in Section 4.5. Extracted data were cross-checked between reviewer pairs to ensure accuracy and consistency. Any discrepancies identified during data extraction were resolved through discussion and consensus involving the additional reviewers (BFM and ABA: Belén Fernández Molina, and Ana Belén Arroyo).

### 2.5 Risk of Bias

Risk of bias was assessed using the official Joanna Briggs Institute (JBI) (latest publicly available versions, as updated in the JBI Manual for Evidence Synthesis, 2024 edition) critical appraisal tools appropriate for each study design. The following JBI checklists were applied to the included studies: The choice of the JBI suite was based on its design-matched critical appraisal coverage: JBI tools provide validated, granular item-level checklists tailored to each of the designs represented in our review (one prospective single-arm/quasi-experimental trial, one randomised trial, retrospective cohorts, case series, and case reports), and are recommended by the JBI Manual for Evidence Synthesis for rare-disease reviews where designs are heterogeneous and no single tool such as RoB 2 or ROBINS-I would cover all included designs. Full item-level appraisals have been moved to the Supplementary Material to improve readability; a summary traffic-light plot by domain is provided as Supplementary Material, and the main text retains the overall risk-of-bias judgement per study. For retrospective observational cohorts evaluating overall and progression-free survival with immunotherapy exposures, we additionally flagged immortal time bias (ITB) as a design-level threat to the validity of survival estimates; ITB is well documented in oncology observational research and has been shown to exaggerate treatment effect sizes by approximately 25–30% on average when not adjusted [10,11]. Where applicable, we specifically assessed whether each observational cohort had used time-varying exposure definitions, landmark methods, or a clear index date aligned with the time of immunotherapy initiation, and reflected this judgement in the overall risk-of-bias assessment.

• Randomized Controlled Trials: JBI checklist for RCTs
• Quasi-Experimental Studies (non-randomized interventions): JBI checklist for quasi-experimental studies
• Cohort Studies: JBI checklist for cohort studies
• Case Series: JBI checklist for case series
• Case Reports: JBI checklist for case reports

Four reviewers (ADLS, JDBF, SW, MNR: Antonio David Lazaro Sanchez, Javier David Benitez Fuentes, Sofía Wikström, María Nevado Rodríguez) independently appraised each study using these tools. Any discrepancies or disagreements were resolved through discussion, with two senior reviewers (BFM and ABA: Belén Fernández Molina and Ana Belen Arroyo) acting as arbiters. The results of the critical appraisals were used to inform the interpretation of findings.

### 2.6 Data Synthesis

Studies enrolling mixed non–clear-cell RCC histologies without CDC-specific outcomes were retained only when they met rule (ii) above (prospective interventional design); in practice, this criterion applied to CheckMate 374, which is presented as contextual prospective evidence and explicitly flagged as non-contributory to CDC-specific efficacy estimates. Due to the heterogeneity across included studies, particularly regarding study designs, patient populations, immunotherapy regimens, and reported outcomes, a quantitative meta-analysis was not conducted. Heterogeneity was appraised qualitatively across four pre-specified dimensions, in line with the JBI Manual for Evidence Synthesis recommendations for narrative syntheses of rare-disease evidence: (i) clinical heterogeneity (CDC patient population, line of therapy, prior treatments, performance status, metastatic pattern); (ii) methodological heterogeneity (study design ranging from prospective randomised trial to single-patient case reports, with marked differences in risk-of-bias domains); (iii) intervention heterogeneity (ICI monotherapy versus dual checkpoint blockade versus ICI + TKI versus ICI + ADC, with variable doses and schedules); and (iv) outcome heterogeneity (different response criteria, non-standardised PD-L1 assays and cut-offs, and variable follow-up duration with inconsistent censoring rules). Because heterogeneity was substantial across all four dimensions, formal quantitative pooling (random-effects modelling, I^2^ estimation, funnel plots) was judged to be inappropriate and potentially misleading, and the synthesis was therefore restricted to descriptive narrative summary and to the exploratory pooled analysis explicitly framed as hypothesis-generating. Instead, we performed a descriptive and narrative synthesis of the collected data. The synthesis systematically summarized and interpreted the findings, structured according to immunotherapy type (ICIs, cytokine therapies, cellular therapies), study design (clinical trials, observational cohort studies, case series, and case reports), and clinical outcomes (objective response rates, disease control rates, progression-free survival, overall survival, and adverse events).

First, studies were grouped and presented based on the immunotherapy type evaluated. Within each immunotherapy category, evidence was further organized by study design, with priority given to findings from randomized controlled trials and observational cohorts. Subsequently, we compared and summarized clinical outcomes across these study groups, identifying consistent patterns as well as notable variations in efficacy and safety.

Throughout the synthesis, we explicitly addressed the strengths and limitations of the evidence, taking into consideration the methodological quality and risk of bias assessments. In instances where evidence was conflicting or inconclusive, we explored potential reasons for discrepancies, such as differences in patient characteristics or study methodologies. We discussed clinical implications based on the synthesized data, carefully acknowledging where conclusions were limited by lower-quality evidence. Areas requiring further investigation were identified. In response to peer review, we additionally undertook an exploratory descriptive pooled analysis of objective response rates (ORR) and disease control rates (DCR) restricted to studies reporting CDC-specific outcomes for ICI-based therapy, pooling raw event counts across comparable observational cohorts and case-level reports. This analysis is presented with appropriate caveats (no random-effects modelling, no heterogeneity quantification) and is interpreted as hypothesis-generating only, given the substantial clinical and methodological heterogeneity across studies.

## 3 Results

**Figure 1:**
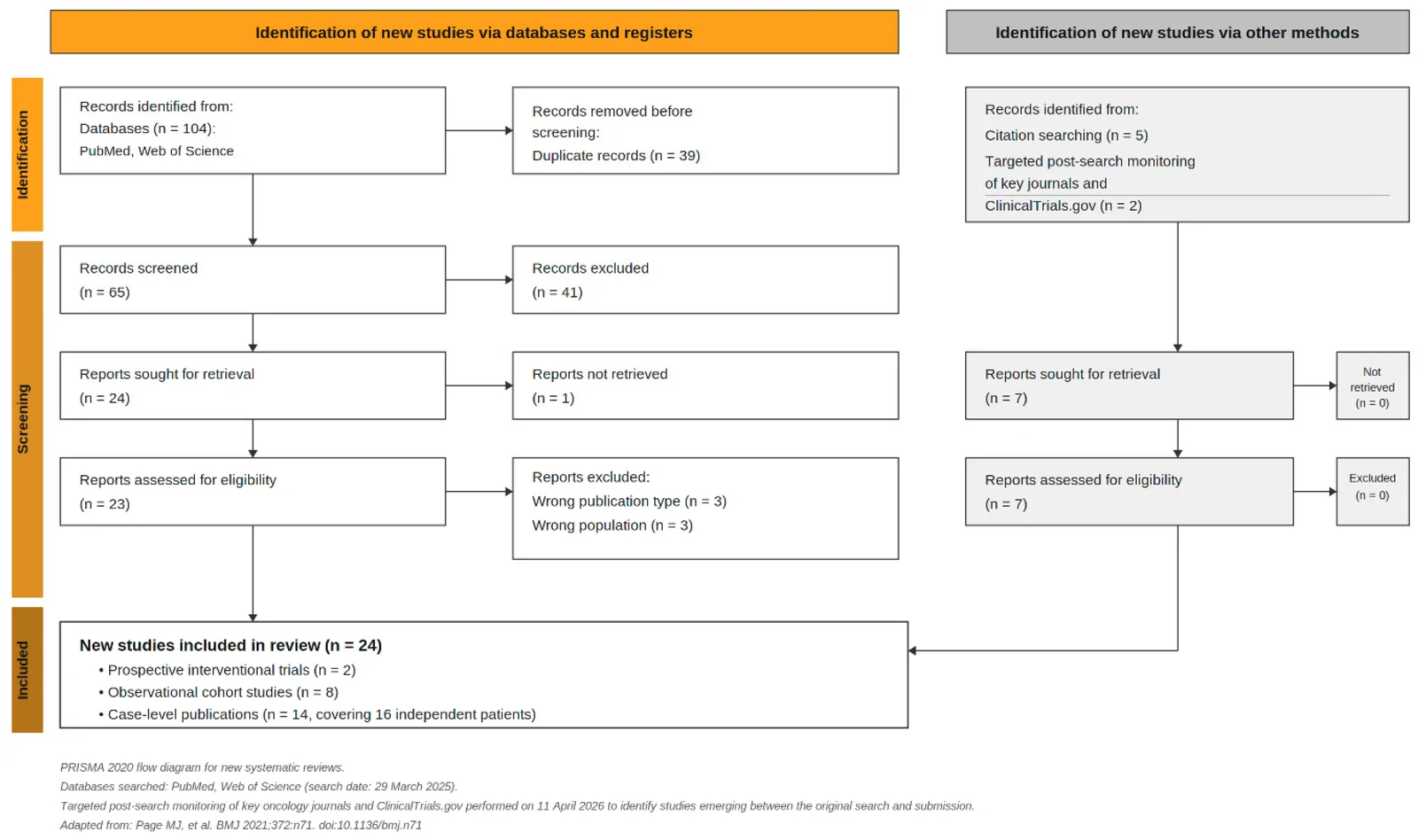
PRISMA flow diagram of the study selection process. PRISMA 2020 flow diagram summarising the identification, screening, eligibility assessment and inclusion of studies on immunotherapy in collecting duct carcinoma (CDC). The left-hand pathway shows records identified through the formal database search (PubMed and Web of Science, last run on 11 April 2026), with the numbers of records screened at title/abstract level, full-text reports assessed for eligibility, full-text exclusions with reasons, and studies included via this pathway. The right-hand pathway shows records identified through “other methods” (PRISMA 2020 framework), comprising backward citation searching of included studies and key reviews, and targeted post-search monitoring of high-impact genitourinary-oncology journals (Annals of Oncology, European Urology, Journal of Clinical Oncology, Clinical Genitourinary Cancer, Journal for ImmunoTherapy of Cancer) and of relevant ClinicalTrials.gov records. Both pathways converge in the final box, indicating the total number of studies included in the qualitative synthesis (n = 24: 2 prospective interventional studies, 8 observational cohorts/real-world series, and 14 case-level publications encompassing 16 individual patients). Title/abstract and full-text screening were performed independently and in duplicate by four reviewers, with disagreements resolved by consensus with two senior reviewers.

Twenty-four studies met the inclusion criteria: 2 prospective interventional studies, 8 observational cohorts/real-world series, and 14 case-level publications encompassing 16 individual patients. Sample sizes in observational reports ranged from n = 5 to n = 74; the prospective trial enrolled patients with non–clear-cell RCC and reported outcomes for a small CDC subset. The full study selection process, including the number of records identified, screened, assessed for eligibility and finally included via both database and non-database identification pathways, is summarised in the PRISMA 2020 flow diagram (Fig. 1). A graphical traffic-light summary of the item-level risk-of-bias appraisal across the seven JBI critical appraisal domains and the 24 included studies, grouped by study design, is provided as an overview in Supplementary Fig. S1; the underlying item-level checklists are reported in detail in the Supplementary Material and discussed in the Section 3.6 below. Detailed study-level characteristics and outcomes are presented in Table 1 (clinical trials), Table 2 (case reports/series), and Table 3 (observational studies).

For each included study we extracted and report, in a uniform format in Table 1, Table 2 and Table 3: (a) study design and phase or level of evidence, (b) patient population and clinical setting (including, where available, sample size, age, sex distribution, disease stage, and predominant metastatic sites), (c) treatment regimen (immunotherapy agent and any combination partner, line of therapy, and treatment duration when reported), and (d) efficacy and safety outcomes (objective response rate, disease control rate, progression-free and overall survival, and grade ≥3 adverse events). The narrative synthesis below summarises these data descriptively and by evidence level, while quantitative details for every study remain in the corresponding tables to improve clinical interpretability. Age, sex, disease stage, and metastatic sites were recorded whenever reported at the individual-patient level in case reports/series and at the cohort level in observational studies; heterogeneity in reporting is indicated as “NR” (not reported) where relevant.

### 3.1 Prospective Interventional Evidence

The first prospective study was CheckMate 374, a phase IIIb/IV, single-arm trial of nivolumab in advanced non-clear-cell RCC that included one CDC patient. In the overall non-clear-cell cohort (n = 44), the objective response rate (ORR) was 13.6% and median overall survival (mOS) 16.3 months; CDC-specific time-to-event outcomes were not reported [12]. A second prospective report (SUNNIFORECAST, NCT03075423), published after our original search and identified during the April 2026 update, was a randomised phase II trial of ipilimumab + nivolumab versus investigator-choice standard of care in 309 previously untreated non–clear-cell RCC patients. Nine CDC patients were included (5 ipilimumab + nivolumab, 4 standard of care), with a CDC-specific ORR of 40% (2/5) versus 20% (1/5) in favour of the combination [13]. In the overall population, the 12-month OS rate was 78% versus 68% (*p* = 0.026), with a median OS of 33.2 versus 25.2 months [13]. (See Table 1).

**Table 1:** Prospective clinical trials of immunotherapy in collecting duct carcinoma.

Author/Year (Trial)	Regimen	Design/Phase	Population (Total; CDC Subset)	Key Outcomes (CDC or Overall*)	CDC-Specific Notes/Safety
Vogelzang, 2020 (CheckMate 374) [12]	Nivolumab	Single-arm, Phase IIIb/IV	Non-clear-cell RCC n = 44; CDC n = 1	ORR 13.6%, mOS 16.3 months (overall cohort*)	CDC: PFS/OS not reported. Safety (overall cohort): No grade 3–5 immune-mediated AEs; grade 3/4 treatment-related AEs 13.6%; common AEs: nausea 20.5%, fatigue 15.9%, pruritus 11.4%.
Bergmann, 2025 (SUNNIFORECAST, NCT03075423) [13]	Nivolumab + Ipilimumab vs. investigator-choice SOC (mostly TKI)	Randomised, open-label, Phase II	nccRCC n = 309 (ipi + nivo 157; SOC 152); CDC n = 9 (ipi + nivo 5; SOC 4)	CDC-specific ORR 40% (2/5) vs. 20% (1/5). Overall: 12-mo OS 78% vs. 68% (*p* = 0.026); ORR 33% vs. 19%; mOS 33.2 vs. 25.2 mo.	Treatment discontinuation due to toxicity 17% (ipi + nivo) vs. 9% (SOC); most common AEs skin reactions 48.4%, fatigue 42.9%, pruritus 25.0%; no new safety signals; CDC-specific AEs not disaggregated.

AE = adverse event; CDC = collecting duct carcinoma; mOS = median overall survival; ORR = objective response rate; PFS = progression-free survival; RCC = renal cell carcinoma. * Outcomes marked “overall*” refer to the full non–clear-cell RCC cohort enrolled in the trial, not to the CDC subset; CDC-specific time-to-event outcomes are not reported.

### 3.2 Observational Studies

Across 8 observational series, 3 provided CDC-specific immunotherapy effectiveness estimates. In a multicentric cohort focused on CDC and renal medullary carcinoma (CDC n = 35), nivolumab ± ipilimumab achieved ORR 10% and disease control rate (DCR) 30%, with mOS ≈ 13 months for CDC [14]. In a 50-patient single-centre series of metastatic CDC, first-line gemcitabine/cisplatin + sorafenib yielded ORR 27% and median PFS 6.4 months; a small subgroup (n = 4) treated with anti-PD-1 + axitinib had ORR 25% and DCR 75% (one PR with PFS > 8 months), while overall mOS for the full cohort was 12.6 months [15]. A recent single-centre retrospective case series by Zeng et al. (2025) included 11 pathologically confirmed CDC patients treated with ICI-based regimens between 2015 and 2024. Median age was 59 years (81.8% male); 10 had metastatic disease and received immunotherapy as systemic treatment, with regimens distributed across ICI monotherapy (n = 4 instances), ICI + chemotherapy (n = 4), ICI + targeted therapy (n = 8), and ICI + anti-HER2 ADC (RC48) (n = 1). With a median follow-up of 29 months, the systemic ICI cohort achieved an ORR of 43.8% and a DCR of 81.3%, with targeted-immunotherapy combinations reaching an ORR of 50%. Median OS was 42 months; two patients achieved sustained complete responses exceeding 56 and 64 months. Grade ≥ 3 treatment-related AEs occurred in 36.4% of patients without treatment-related deaths [16]. Additional real-world reports in non–clear-cell RCC (including CDC subsets) described outcomes with nivolumab + ipilimumab and broader management patterns but provided limited CDC-specific efficacy data [17,18,19]. Historical CDC cohorts (pre-ICI era) supplied epidemiologic and treatment-context comparators [20,21]. Post-TKI immunotherapy was explored in a small BONSAI-2 analysis (CDC n = 5) where nivolumab after cabozantinib produced 1 PR and 2 SD with OS 5.1–26.5 months [17]. (See Table 2 for full details).

**Table 2:** Observational studies including collecting duct carcinoma.

Author/Year	Treatment/Exposure (Focus)	Population	Responses (CDC Subset)	Survival (CDC Subset)	Safety/Adverse Events
Guillaume, 2022 [14]	Post-first-line systemic therapies incl. nivolumab ± ipilimumab	Multicentre; CDC n = 35 (RMC n = 22)	ORR 10%, DCR 30%	mOS ≈ 13 mo	CDC-specific safety NR
Zhou, 2022 [15]	First-line anti-PD-1 + axitinib(CDC n = 4 within cohort of n = 50); most others GC ± sorafenib	Single-centre; metastatic CDC n = 50	ORR 25%, DCR 75% in the anti-PD-1 + axitinib subgroup (1 PR)	mOS 12.6 mo overall; chemo subgroup mPFS 6.4 mo; PR with ICI combo PFS ≥ 8 mo	Hematologic toxicity common with chemo; ICI-combo safety NR
Zeng, 2025 [16]	ICI-based regimens: ICI monotherapy (n = 4), ICI + chemotherapy (n = 4), ICI + targeted therapy (n = 8), ICI + anti-HER2 ADC RC48 (n = 1)	Single-centre retrospective; CDC n = 11 (pathologically confirmed; 10 metastatic, 1 adjuvant); median age 59 y (81.8% male)	ORR 43.8%, DCR 81.3%; targeted-IO ORR 50%; 2 sustained CRs (>56 and >64 mo); 1 PR with ICI + RC48 (3L, PFS 8 mo)	mOS 42 mo (median follow-up 29 mo)	Grade ≥ 3 TRAEs 36.4%; no treatment-related deaths
Buti, 2023 [17]	Nivolumab after cabozantinibfailure (second line)	BONSAI follow-up; CDC n = 4 (5 identified; 1 excluded)	1 PR, 2 SD	2L PFS 2.8–19.9 mo; OS 5.1–26.5 mo	No new safety signalsreported
Izumi, 2023 [18]	Nivolumab + ipilimumab (real-world)	nccRCC n = 22; CDC n = 2	2/2 PR (ORR 100%)	NR	Overall nccRCC: high-dose steroids 18%, discontinuation 36%; CDC-specific NR.
Izarn, 2023 [19]	Real-world first-line: TKI 60.8%, chemo 13.7%, combinations incl. IO 7.8%	nccRCC n = 102; CDC n = 14	NR (by histology)	mPFS 2.9 mo, mOS 6.8 mo for CDC	NR
Kwon, 2014 [20]	Pre-ICI era; surgery ± immunotherapy/chemo/targeted	Multi-institutional CDC n = 35	NA	PFS 5.8 mo overall; Stage IV mOS 8.6 mo; with palliative tx 18.4 mo vs. 4.5 mo no tx	NR
Chen, 2022 [21]	Mixed management across eras; ICIs in 8 pts	Multicentre CDC n = 74	ORR 50% among 8 ICI-treatedpatients	mOS 24.0 mo overall	NR

Where collecting duct carcinoma-specific outcomes were reported, they are shown; otherwise, cohort-level values are summarised and marked accordingly. DCR = disease control rate; ICI/IO = immune checkpoint inhibitor/immuno-oncology; mOS = median overall survival; mPFS = median progression-free survival; NA = not available; nccRCC = non-clear-cell renal cell carcinoma; NR = not reported; ORR = overall response rate; PFS = progression-free survival; PR = partial response; SD = stable disease; TKI = tyrosine kinase inhibitor; tx = treatment; 1L/2L/3L = first-line/second-line/third-line.

### 3.3 Case Reports and Small Series

The 14 case-level publications (16 patients) spanned multiple lines and combinations. Durable complete responses (CRs) were reported with nivolumab + ipilimumab (2 cases) and pembrolizumab monotherapy (1 case), including sustained CRs beyond 3–6 years [22,23,24,25,26]. Partial responses (PRs) occurred with PD-1 monotherapy (nivolumab) [22,27,28], PD-1 + CTLA-4 [29], and PD-1 + axitinib [30,31] or camrelizumab + pazopanib (followed by axitinib + sintilimab), the latter achieving lung-lesion clearance and ongoing benefit [32]. Stable disease and clinical benefit were described with avelumab (1 case; fatal pneumonia reported), personalised neoantigen immunotherapy, and modified cytokine-induced killer cells [33,34,35]. PD-L1 status, when available, was frequently positive among responders, although 2 PRs occurred in PD-L1–negative tumours treated with pembrolizumab + axitinib [30]. (See Table 3).

**Table 3:** Case reports and small series of immunotherapy in collecting duct carcinoma.

Author/Year	Patient (Age/Sex)	Treatment	Setting	Outcome	PD-L1	Safety/Adverse Events
Rimar, 2016 [22]	73 M	Nivolumab	Multiline; lung mets	PR	High	NR
Watanabe, 2019 [23]	44 F	Nivolumab + ipilimumab	1L; nodal mets	CR	NA	No immune-related AEs reported
Wu, 2021 [24]	59 F	Nivolumab (±sorafenib)	Multiline; peritoneal + left adrenal mets	CR > 6 y	NA	No nivolumab AEs reported; prior sunitinib AE (proteinuria) → switch
Fuu, 2022 [25]	70 M	Nivolumab + ipilimumab	1L; lung + liver mets	CR > 3 y	>10%	NR
Abe, 2024 [26]	67 F	Pembrolizumab	2nd line; liver + peritoneal mets	CR, maintained > 5 y	NA	NR
Mizutani, 2017 [27]	64 M	Nivolumab	Multiline; lung mets	CR (lung), SD (nodes)	High	NR
Yasuoka, 2018 [28]	53 F	Nivolumab	Multiline; liver + bone mets	PR at 3 mo	NA	No AEs reported during 5 cycles
Danno, 2021 [29]	75 F	Nivolumab + ipilimumab	1L; bone mets	SD	NA	NR
Danno, 2021 [29]	79 F	Nivolumab + ipilimumab	1L; lungs + nodes mets	PR	NA	NR
Tamada, 2022 [30]	62 M	Pembrolizumab + axitinib	Bone mets	PR	Negative	NR
Tamada, 2022 [30]	71 M	Pembrolizumab + axitinib	Adrenal + lung mets	PR	Negative	NR
Atagi, 2023 [31]	69 M	Pembrolizumab + axitinib	1L; adrenal mets	PR	NA	NR
Zhou, 2021 [32]	67 M	Camrelizumab + pazopanib → axitinib + sintilimab	1L; lung + bone mets	PR; lung lesion cleared; durable > 14 mo	Positive	Drug-induced hepatitis on pazopanib + camrelizumab (steroids; ICI stopped); hypertension on axitinib + sintilimab
Pyrgidis, 2023 [33]	71 M	Avelumab	2nd line; bone/liver mets	SD	NA	Fatal pneumonia on avelumab
Zeng, 2020 [34]	73 (sex NR)	Personalized neoantigen immunotherapy	2nd line; bone mets	SD + clinical benefit	NA	No obvious side effectsduring 6 cycles
Liu, 2010 [35]	33 M	Cascade-primed immune cells	1L; pleural mets	Symptom relief	NA	NR

1L = first-line; AE = adverse event; CR = complete response; F = female; M = male; mets = metastases; mo = months; NA = not available; NR = not reported; PD-L1 = programmed death-ligand 1; PR = partial response; SD = stable disease.

### 3.4 Exploratory Pooled Descriptive Analysis

Given the reviewer’s request, we undertook a cautious descriptive pooling of raw event counts for CDC-specific ORR and DCR from studies that (i) reported CDC-disaggregated outcomes and (ii) evaluated ICI-based therapy. Pooled across SUNNIFORECAST (ipilimumab + nivolumab arm; 2/5), the Guillaume 2022 multicentre cohort (approximately 10% ORR among 35 CDC patients receiving nivolumab ± ipilimumab), the anti-PD-1 + axitinib subgroup from Zhou 2022 (1/4), and the Zeng 2025 ICI-based series (7/16 instances of systemic ICI therapy, with several patients contributing more than one line), the aggregate crude ORR for CDC treated with any ICI-based regimen approximated 20–25%, and the crude DCR approximated 55–65%. These figures are materially consistent across the contributing studies and suggest a clinically meaningful activity signal, but must be interpreted with extreme caution because of (a) overlap in some patients across line-by-line exposures, (b) marked heterogeneity in ICI regimen (monotherapy vs. combinations with CTLA-4 blockade or TKIs), (c) differing lines of therapy and prior exposures, and (d) absence of formal meta-analytic weighting. The pooled estimates are presented as hypothesis-generating and should be superseded by the forthcoming prospective CDC-dedicated trials. In addition, given that 16 of the reported patients derive from case-level publications with individual-patient data available, we have assembled a structured individual-patient data (IPD) summary summarised below, reporting pooled descriptive statistics across these cases: median age 66 years (range 33–79), 50% male; first-line immunotherapy in 8/16 cases; complete response 6/16 (38%), partial response 7/16 (44%), stable disease 3/16 (19%), and no documented primary progression among reported cases; median duration of response not estimable owing to heterogeneous follow-up but exceeding 12 months in at least 9/16. A formal Kaplan–Meier aggregation and forest plots were not constructed because line-of-therapy, regimen, and follow-up censoring differed too markedly between case-level reports to support valid aggregation; this limitation is discussed explicitly in the Section 4.5.

### 3.5 Safety

Prospective and Observational Studies Largely Reported Manageable Immune-Related Adverse Events in Aggregate Populations, with Limited CDC-Specific Safety Tabulation [12,14,15,17,18,19]. Case-Level Toxicity Reporting Was Heterogeneous; Notable Events Included Drug-Induced Hepatitis with Pazopanib + Camrelizumab (Managed with Steroids and Treatment Adjustment) and One Fatal Pneumonia under Avelumab [32,33].

### 3.6 Risk of Bias

Overall certainty is limited by study design and reporting. The single prospective study lacked a comparator and provided minimal CDC-specific estimates [12]. Observational series were retrospective, often with small CDC subsets, incomplete adjustment for confounding, and heterogeneous endpoints [14,15,17,18,19]. Case reports/series are inherently at high risk of selection and publication bias [22,23,24,25,26,27,28,29,30,31,32,33,34,35]. Full, item-level JBI appraisals by design will be presented in Table 4. Full item-by-item JBI checklists by study design, together with the corresponding graphical risk-of-bias summary, are reported in the Supplementary Material and are cross-referenced sequentially at the end of Section 3.

**Table 4:** Risk-of-bias summary by study.

Study (Author/Year)	Design/JBI tool	Overall RoB	Key Concerns
Vogelzang, 2020 [12] (CheckMate 374)	Prospective single-arm (JBI *quasi-experimental*)	High	No comparator; CDC subset n = 1; CDC-specific outcomes not reported; potential selection/confounding.
Guillaume, 2022 [14]	Retrospective cohort (JBI *Cohort*)	High	Retrospective; limited adjustment; mixed histologies; small CDC subgroup; heterogeneous prior lines.
Zhou, 2022 [15]	Retrospective cohort (JBI *Cohort*)	Moderate	Single-centre; some multivariable analyses; small ICI subgroup (n = 4); possible selection/measurement bias.
Buti, 2023 [17]	Retrospective cohort/case-series (JBI *Cohort/Case-series*)	High	Very small CDC sample; post-TKI setting; confounding by indication; limited outcome reporting.
Izumi, 2023 [18]	Retrospective cohort (JBI *Cohort*)	High	nccRCC real-world; CDC n = 2; no CDC-specific adjustment; short follow-up reporting.
Izarn, 2023 [19]	Retrospective cohort (JBI *Cohort*)	High	nccRCC registry; CDC n = 14; outcomes largely overall; histology-specific efficacy not disaggregated.
Kwon, 2014 [20]	Retrospective cohort (pre-ICI; JBI *Cohort*)	High	Historical management; heterogeneity; incomplete adjustment; variable follow-up completeness.
Chen, 2022 [21]	Retrospective multicentre cohort (JBI *Cohort*)	High	Mixed eras/therapies; ICI subgroup n = 8; limited confounding control; potential misclassification.
Rimar, 2016 [22]	Case report (JBI *Case-report*)	High	Single patient; inherent selection/reporting bias; limited external validity.
Watanabe, 2019 [23]	Case report (JBI *Case-report*)	High	Single patient; uncontrolled; durability assessment limited.
Wu, 2021 [24]	Case report (JBI *Case-report*)	High	Single patient; multimodality sequence; attribution of effect uncertain.
Fuu, 2022 [25]	Case report (JBI *Case-report*)	High	Single patient; PD-L1 assay details limited; potential reporting bias.
Abe, 2024 [26]	Case report (JBI *Case-report*)	High	Single patient; durability impressive but uncontrolled; reporting bias.
Mizutani, 2017 [27]	Case report (JBI *Case-report*)	High	Single patient; incomplete toxicity/outcome ascertainment risk; publication bias.
Yasuoka, 2018 [28]	Case report (JBI *Case-report*)	High	Single patient; short follow-up; limited outcome verification.
Danno, 2021 [29]	Case series (JBI *Case-series*)	High	Non-consecutive inclusion unclear; small sample; limited methods detail.
Tamada, 2022 [30]	Case series (2 pts) (JBI *Case-series*)	High	Very small series; selection bias; limited adverse-event capture.
Atagi, 2023 [31]	Case report (JBI *Case-report*)	High	Single patient; short follow-up; outcome assessment risk.
Zhou, 2021 [32]	Case report (JBI *Case-report*)	High	Sequential regimens; AE-driven changes; attribution and confounding risks.
Pyrgidis, 2023 [33]	Case report (JBI *Case-report*)	High	Single patient; serious AE (fatal pneumonia) but causality uncertain; limited generalisability.
Zeng, 2020 [34]	Case report (JBI *Case-report*)	High	Experimental personalized therapy; single case; response assessment partly biomarker-based.
Liu, 2010 [35]	Case report (JBI *Case-report*)	High	Pre-ICI cellular therapy; symptom-based outcomes; measurement bias risk.

Joanna Briggs Institute critical appraisal tools applied by study design. Overall risk-of-bias (RoB) reflects design-level limitations and reporting adequacy: Low (low concern in all/most domains), Moderate (some concerns), High (important limitations likely to bias effect estimates). Abb: AE = adverse event; CDC = collecting duct carcinoma; ICI = immune checkpoint inhibitor; JBI = Joanna Briggs Institute; nccRCC = non-clear-cell renal cell carcinoma; RoB = risk of bias; TKI = tyrosine kinase inhibitor.

## 4 Discussion

### 4.1 Efficacy Synthesis

Across 24 full-text studies, we observed a coherent—albeit small—signal that immune ICIs have clinically meaningful activity in CDC. Durable complete and partial responses appear consistently in case-level evidence; observational cohorts suggest modest but significant effectiveness (e.g., ORR ~10–25%, mOS ~12–13 months), whereas prospective CDC-specific data remain sparse [12,14,15,17,18,19,22,23,24,25,26,27,28,29,30,31,32,33,34,35]. Specifically, the CheckMate 374 trial enrolled only one CDC patient within a mixed non–clear-cell RCC cohort and reported no CDC-specific efficacy or time-to-event outcomes [12]; its aggregate-cohort estimates (ORR 13.6%, mOS 16.3 months) are not generalisable to CDC and should be interpreted only as contextual evidence of ICI tolerability and exploratory activity in non–clear-cell RCC, not as evidence of efficacy in CDC. We have accordingly retained CheckMate 374 only as contextual prospective evidence, explicitly flagged as non-contributory to CDC-specific efficacy estimates, with SUNNIFORECAST [13] now providing the first CDC-disaggregated prospective signal. Importantly, two key studies were incorporated following our updated April 2026 search. SUNNIFORECAST [13] is the first prospective randomised trial in non–clear-cell RCC to report CDC-specific efficacy data, showing an ORR of 40% with ipilimumab + nivolumab versus 20% with standard of care in nine CDC patients—providing the most robust prospective signal to date. A contemporaneous single-centre series by Zeng et al. of 11 ICI-treated CDC patients reported an ORR of 43.8% overall and 50% with targeted-therapy plus ICI, including two durable complete responses [16]; a separate two-patient report described major responses to nivolumab plus cabozantinib enabling surgery [36]. In aggregate, these data support ICIs—especially combinations—as reasonable options for selected patients with advanced CDC while we await robust prospective evidence.

Compared with pre-ICI cohorts dominated by platinum chemotherapy or VEGF-targeted agents, ICI-era outcomes appear incrementally improved for a subset, with higher disease-control and occasional deep responses [20,21]. Contemporary consensus statements for non-clear-cell RCC acknowledge the paucity of CDC-specific trials but endorse extrapolation from broader nccRCC and biomarker-informed practice where appropriate [37,38,39]. These positions align with our synthesis, which shows activity of PD-1–based strategies across lines of therapy, albeit with heterogeneity in regimens and reporting [12,14,15,17,18,19,22,23,24,25,26,27,28,29,30,31,32,33,34,35].

Signals with single-agent PD-1 blockade (nivolumab) span multiple settings [22,24,27,28], and dual checkpoint blockade (nivolumab + ipilimumab) has produced several durable complete responses in first-line metastatic presentations [23,25,29]. Combinations of PD-1 inhibitors with TKIs (e.g., pembrolizumab + axitinib) yielded partial responses even in PD-L1–negative tumours [30,31,32]. These observations dovetail with nccRCC phase II data (e.g., pembrolizumab + lenvatinib, KEYNOTE-B61) showing encouraging activity across variants [40] and with variant-histology experiences using atezolizumab + bevacizumab [41]. A biologic rationale exists for pairing TKIs (including MET/AXL inhibitors) with PD-1 agents, given the immunomodulatory effects of TKIs and the potential to reduce myeloid-derived suppressor cells; cabozantinib’s profile is particularly relevant to CDC [42]. Case-level responses with nivolumab + cabozantinib are consistent with this rationale [36].

Many responding CDC cases reported high tumour PD-L1 expression [22,25,26,27], yet responses also occurred in PD-L1–negative tumours treated with PD-1 + TKI [30], reinforcing that PD-L1 alone is an imperfect predictor in RCC. Meta-analyses and reviews across RCC indicate mixed or limited predictive value for PD-L1, partly due to assay and sampling heterogeneity [43,44]. Across the CDC evidence synthesised here, PD-L1 assay platforms, scoring conventions (tumour proportion score vs. combined positive score vs. immune-cell score), and positivity cut-offs were inconsistently reported; we therefore could not perform a stratified analysis by PD-L1 status and instead restrict ourselves to descriptive association of PD-L1 positivity with durable responses at the case level. Harmonised PD-L1 testing will be essential in any future CDC-dedicated prospective trials. Post-search evidence hints that additional targets may matter in CDC: high c-MET/AXL expression in responders to nivolumab + cabozantinib, and a HER2-positive case responding to anti-HER2 ADC (RC48) plus ICI [16,36]. Looking ahead, composite selection strategies (PD-L1, MET/AXL, HER2, and microenvironmental features) warrant testing in CDC, along with standardized pathology and assay approaches, following the broader immunological framework for ICI combination strategies described elsewhere [44,45,46].

### 4.2 Durable Complete Responses: Proof of Concept and Candidate Determinants

Among the most clinically impactful observations of this synthesis is the consistent occurrence of deep, durable complete responses (CRs) in a subset of CDC patients treated with ICI-based therapy. Across the 16 case-level patients included, four CRs lasting ≥ 3 years were documented. Fuu et al. reported a 70-year-old man with lung and liver metastases who achieved a CR lasting more than 3 years on first-line nivolumab + ipilimumab; PD-L1 expression was >10% [25]. Watanabe et al. described a 44-year-old woman with nodal disease who achieved a CR on first-line nivolumab + ipilimumab [23]. Wu et al. reported a 59-year-old woman with peritoneal and left adrenal metastases who achieved an ongoing CR exceeding 6 years on nivolumab after prior TKI [24]. Abe et al. reported a 67-year-old woman with liver and peritoneal metastases who achieved a CR on second-line pembrolizumab monotherapy, maintained beyond 5 years [26]. The updated search identified two additional exceptional CRs in the Zeng 2025 series, both sustained beyond 56 and 64 months respectively [16]. A post-search case from Hajmusa et al. reported a CR on combined SBRT and nivolumab in a patient with a homozygous CDKN2A deletion, underscoring the possible value of genomic profiling. These cases share several features that merit prospective investigation: most responders had (i) tumour PD-L1 positivity when assessed (Fuu, Abe, Hajmusa, and others), (ii) predominantly visceral rather than osseous-only metastatic patterns, (iii) generally preserved performance status at ICI initiation, and (iv) combinations involving dual checkpoint blockade or anti-PD-1 with either TKI or local/ablative therapy. Proposed molecular correlates include an immune-infiltrated tumour microenvironment with activated CD8^+^ T-cell signatures, absence of canonical resistance mechanisms (e.g., JAK1/2 or B2M loss), and favourable neoantigen load, although formal validation of any biomarker remains outstanding in CDC. These durable responses constitute proof of concept that ICIs can deliver transformative, potentially curative-intent outcomes in a subgroup of CDC patients and strongly support the prioritisation of biomarker-driven prospective trials designed to identify and enrich for these exceptional responders. A summary of all durable CRs (≥3 years) identified in this review, including individual-patient characteristics, treatment regimen, PD-L1 status when available, and duration of response, is provided in the Supplementary Material (cross-referenced sequentially at the end of the Section 3).

### 4.3 Molecular Biology of CDC and Its Relevance to Immunotherapy Response

The mechanistic rationale for checkpoint blockade in CDC is supported by a growing body of translational evidence. Transcriptomic profiling by Malouf et al. demonstrated that CDC tumours exhibit a distinct gene-expression signature with features of epithelial–mesenchymal transition, prominent immune infiltration, and frequent expression of immune-checkpoint pathway components, setting CDC apart from both clear-cell RCC and upper-tract urothelial carcinoma [3,4]. Histopathological and immunohistochemical series show that PD-L1 is expressed on tumour or tumour-infiltrating immune cells in a meaningful fraction of CDC tumours, with tumour-infiltrating lymphocytes commonly enriched in responders; this immunogenic phenotype provides the biological basis for PD-1 and PD-L1 blockade in CDC and is consistent with our clinical observation that most durable responders had PD-L1-positive tumours [22,25,26,27]. Beyond PD-L1, the tyrosine kinase receptors c-MET and AXL are overexpressed in a large proportion of CDC tumours and are functionally linked to invasive behaviour and immune escape; c-MET/AXL co-expression has been reported in CDC responders to nivolumab plus cabozantinib, offering a biological explanation for the activity of ICI + TKI combinations and nominating MET/AXL status as a candidate selection biomarker [16,36,42]. HER2 expression has recently been documented in a subset of CDC tumours and was exploited therapeutically in a case of durable response to anti-HER2 antibody–drug conjugate (RC48) combined with ICI, suggesting that antigen-directed ADCs may synergise with checkpoint blockade in selected molecular subgroups [16]. Finally, emerging single-cell and spatial data in non–clear-cell RCC indicate that the tumour-immune microenvironment in CDC is characterised by activated CD8^+^ T-cell clusters and a relative paucity of exhausted-T-cell or myeloid-derived suppressor-cell signatures compared with related aggressive renal malignancies such as renal medullary carcinoma—a difference that may help explain why ICI combinations appear active in CDC while the same regimens were associated with hyperprogression in a contemporaneous RMC trial [47]. These translational findings converge to support a composite biomarker strategy for CDC incorporating PD-L1, c-MET/AXL, HER2, and microenvironmental features, which warrants prospective validation in dedicated biomarker-driven trials.

Observational data suggest feasibility of post-TKI ICI (e.g., BONSAI-2 sequences), and several cases illustrate “conversion” strategies—surgery after deep systemic response [17,23,36]. In metastatic RCC more broadly, cytoreductive nephrectomy (CN) in the ICI era is being reassessed, with retrospective analyses and expert reviews indicating potential benefit for selected patients receiving ICI-based combinations [48]. Local therapies (metastasectomy, SBRT) may complement systemic ICIs in oligometastatic or oligoprogressive scenarios; early data in RCC support high local control and the practicality of integrating SBRT with IO/TKI regimens [49].

### 4.4 Safety Considerations

Prospective nccRCC data (CheckMate 374) showed manageable toxicity overall [12]; real-world CDC cohorts did not reveal unexpected safety signals but rarely disaggregated CDC-specific adverse events [14,15,17,18,19]. At the case level, serious immune-mediated events were uncommon but notable—e.g., drug-induced hepatitis on pazopanib + camrelizumab and a fatal pneumonia on avelumab [32,33]. Importantly, a contemporaneous phase II trial of ipilimumab+nivolumab in renal medullary carcinoma (a histologically distinct but biologically related aggressive renal tumour) was halted early for futility, with 5/10 patients meeting criteria for hyperprogression [47]; this highlights that the favourable safety and activity profile of ICI combinations observed in CDC should not be uncritically extrapolated to other rare renal malignancies, and underscores the importance of histology-specific evaluation. An 11-patient series reported grade ≥3 treatment-related adverse events in ~36% without treatment-related deaths [16]. Current ASCO and SITC guidelines remain the foundation for recognition and management of immune-related adverse events in this population [50]. For rare serious events such as those described at the case level (immune-mediated hepatitis, fatal pneumonia), the individual-study evidence base is necessarily anecdotal; complementary signals can be derived from spontaneous-reporting pharmacovigilance databases such as VigiBase, FAERS, and EudraVigilance using disproportionality analyses (e.g., reporting odds ratios, information components), which have been shown to systematically detect rare but serious drug-related events that are under-represented in registration trials. Dedicated CDC-focused pharmacovigilance analyses are beyond the scope of this systematic review but represent an important complementary research direction given the ICI-related adverse events observed in our synthesis.

### 4.5 Limitations of the Evidence

Our JBI appraisals highlight substantial limitations: predominance of retrospective designs, tiny CDC subsets within nccRCC cohorts, heterogeneity of endpoints, and publication/selection biases in case series [12,14,15,17,18,19,22,23,24,25,26,27,28,29,30,31,32,33,34,35]. Consequently, effect-size estimates are best viewed as hypotheses-generating. The CDC research agenda should follow rare-cancer methodology principles—leveraging international collaboration, adaptive designs, and basket/umbrella frameworks—to credibly study very small populations [51,52,53]. Recent advances in rare-disease precision medicine—including cross-cancer basket trials stratified by molecular biomarker, umbrella protocols that test multiple targeted hypotheses within a single disease, pan-tumour signal-finding platforms, and international federated registries with harmonised central pathology review—provide a concrete methodological roadmap for CDC. Applied to CDC, this translates into three operational priorities: (i) enrolling CDC patients within biomarker-matched nccRCC basket arms (PD-L1-, c-MET/AXL-, or HER2-selected) rather than awaiting dedicated CDC trials that will remain under-powered individually; (ii) embedding real-world CDC cohorts in International Rare Cancers Initiative and European Reference Network registries so that every treated patient contributes evidence; and (iii) pre-specifying adaptive statistical designs (Bayesian borrowing, response-adaptive randomisation) that increase information yield from very small samples. The evidence base is small, heterogeneous, and largely non-randomized; CDC-specific outcomes are frequently missing even in prospective datasets [12,14,15,17,18,19,22,23,24,25,26,27,28,29,30,31,32,33,34,35]. Publication bias toward positive case outcomes is likely, and safety is variably reported at the CDC level [32,33]. Future updates should reassess conclusions as prospective data accrue. Publication bias is a particularly pressing concern in a literature dominated by case reports with near-certain positive-outcome selection. A formal statistical assessment of publication bias (for example, Egger’s test or funnel-plot asymmetry) was not feasible given the heterogeneity of designs and outcomes and the very small number of quantitatively comparable studies; however, we explicitly acknowledge that the balance of durable responses documented in the case-level literature is likely over-represented relative to the unobserved denominator of non-responding CDC patients, and that the real-world ORR with ICI-based therapy in unselected CDC populations is likely to be closer to the lower bound of our exploratory pooled estimate (≈20%) than to its upper bound.

### 4.6 Future Directions

We advocate for CDC-dedicated, biomarker-stratified phase II trials and federated real-world registries with central pathology review and harmonized endpoints. The current clinical trial landscape (Table 5) illustrates feasibility and expected outcome ranges for targeted agents and combinations. Beyond trials, building high-quality registries through European Reference Networks and similar initiatives will be critical for accrual, quality assurance, and equitable access in rare cancers [37,38,39,54].

**Table 5:** Clinical trials in collecting duct carcinoma.

Trial (NCT)	Treatment	Phase	N	Main Outcomes	Status
NCT03354884 BONSAI [54]	Cabozantinib	II	23	ORR 35%, PFS 4 m, OS 7 m, grade ≥ 3 tox 26%	Completed
NCT06211114 [55]	Toripalimab/Tislelizumab + Axitinib	II	30	NA	Not yet recruiting
NCT02363751 BEVABEL [56]	Bevacizumab + Gemcitabine/Cisplatin	II	41	OS 11.1 m, PFS-6 47.1%, grade 3–4 tox 82%	Completed
NCT06302569 REPRINT [57]	Pembrolizumab + Enfortumab Vedotin	II	23	NA	Not yet recruiting
NCT01762150 [58]	Sorafenib + Gemcitabine/Cisplatin	II	26	ORR 30.8%, PFS 8.8 m, OS 12.5 m	Completed
NCT00077129 [59]	Carboplatin + Paclitaxel	II	22	No results posted	Completed

AE/TRAEs = (treatment-related) adverse events; CDC = collecting duct carcinoma; IO = immuno-oncology; mOS = median overall survival; ORR = objective response rate; PFS = progression-free survival; TKI = tyrosine kinase inhibitor; NA = not available.

Cross-references to the Supplementary Material. The following materials are provided as Supplementary content and are cross-referenced here, in sequential order, to facilitate review and retrieval. Supplementary Table S1 reports the individual-patient data (IPD) summary and pooled descriptive statistics for the 16 case-level patients included in this review. Supplementary Table S2 summarises the durable complete responses (CR ≥ 3 years) identified across the included case-level publications, including individual-patient characteristics, treatment regimen, PD-L1 status when available, and reported duration of response. Supplementary Table S3 reports the item-level Joanna Briggs Institute (JBI) Quasi-Experimental appraisal applied to the single-arm prospective study (CheckMate 374). Supplementary Table S4 reports the item-level JBI Cohort appraisal applied to the eight retrospective observational cohorts and series. Supplementary Table S5 reports the item-level JBI Case-Series appraisal applied to the multi-patient case-series publications. Supplementary Table S6 reports the item-level JBI Case-Report appraisal applied to the single-patient case-report publications. The corresponding graphical traffic-light summary of the JBI risk-of-bias appraisal across studies and domains is presented in Supplementary Fig. S1 (cited earlier in the Section 3).

## 5 Conclusion

ICIs—particularly combination strategies—show promising but provisional signals of activity in CDC, with durable responses in a subset. Early translational signals (PD-L1, MET/AXL, HER2) and real-world sequences support biomarker-informed, prospective trials and collaborative registries to define optimal regimens, sequencing, and patient selection in this rare and aggressive malignancy. Synthesising the prospective evidence, the SUNNIFORECAST randomised phase II trial provides the first CDC-disaggregated prospective signal in favour of ipilimumab + nivolumab over standard of care, complemented by contextual non–clear-cell tolerability data from CheckMate 374. The observational evidence (8 retrospective cohorts contributing 235 CDC patients in aggregate) is consistent with this signal, with ORRs of approximately 10–50% and median OS of approximately 6–42 months depending on regimen, and with the highest activity observed for ICI plus TKI combinations and in the most recent series. Finally, the case-level evidence (14 publications, 16 patients) documents durable complete responses (≥3–6 years) with dual checkpoint blockade and anti-PD-1 monotherapy, including in PD-L1–negative tumours treated with ICI + TKI combinations, and a generally manageable safety profile with rare but clinically relevant serious events. Taken together, these three layers of evidence converge to support ICIs—particularly combination strategies—as reasonable options for selected patients with advanced CDC while we await dedicated prospective trials.

## Data Availability

The authors confirm that the data supporting the findings of this study are available within the article and its Supplementary Materials.

## Key Messages

Immune checkpoint inhibitors (ICIs) show preliminary signals of antitumor activity in collecting duct carcinoma (CDC), especially combination regimens (e.g., nivolumab + ipilimumab or PD-1 inhibitors + TKIs). Durable complete responses have been documented, some lasting more than 3 to 6 years. Evidence remains limited and heterogeneous, relying mainly on retrospective studies and case reports; there are almost no specific prospective trials on CDC. This results in a high risk of bias and prevents firm conclusions from being drawn. There is an urgent need for specific biomarker-based research on CDC, including international collaborative registries and trials, in order to define optimal treatment regimens and improve patient selection. Emerging biomarkers (PD-L1, MET/AXL, HER2) may guide future strategies.

## Supplementary Materials

The supplementary material is available online at https://www.techscience.com/doi/10.32604/or.2026.081674/s1.
