# Supplementary material for: Immunotherapy in Bellini Duct Carcinoma: A Systematic Review
Source: Oncol Res. 2026 Sep 14;34(10):2. doi: 10.32604/or.2026.081674 (PMC13591354; doi:10.32604/or.2026.081674)
Supplement: Supplementary file 1 [file OncolRes-34-81674-s001.zip › OR-81674_Supplementary_Figure_S1.docx]

**Supplementary Figure S1.** **Traffic-light summary of risk-of-bias judgements by study and Joanna Briggs Institute (JBI) domain.**

**Figure legend.** Graphical traffic-light summary of the item-level risk-of-bias appraisal for the 24 studies included in this systematic review of immune checkpoint inhibitor (ICI)-based therapy in collecting duct carcinoma (CDC). Studies are grouped by design — prospective (top), observational cohorts/series (middle), and case-level publications (bottom) — and listed chronologically within each group. Columns correspond to the seven Joanna Briggs Institute (JBI) critical appraisal domains common to the four checklists applied in this review (RCT, quasi-experimental, cohort, case-series, and case-report tools): D1, selection of participants; D2, definition / measurement of the intervention or exposure; D3, validity and reliability of the outcome measurement; D4, identification and adjustment for confounders or comparability of groups; D5, completeness and duration of follow-up; D6, appropriateness of the statistical analysis; D7, completeness and transparency of reporting. The right-most column (“Overall”) shows the overall risk-of-bias judgement per study, derived by integrating the item-level appraisals using the JBI design-matched algorithm described in the Methods section. Cells are colour-coded as: green plus = Yes (low risk of bias, item adequately addressed and clearly reported); amber question mark = Unclear / some concerns (item partially addressed or insufficiently reported to permit a definitive judgement); red minus = No (high risk of bias, item not addressed, inadequately addressed, or reported in a way that introduces a substantial threat to validity); grey cross = Not applicable (domain not assessable in single-patient case reports — e.g., confounding adjustment or formal statistical analysis). For retrospective observational cohorts evaluating overall and progression-free survival with time-varying immunotherapy exposures, the assessment additionally incorporated immortal time bias as a design-level threat to validity. Each circle reflects the consensus judgement of two pairs of reviewers using the JBI critical appraisal tool matched to study design: RCT for SUNNIFORECAST [13]; Quasi-Experimental for CheckMate 374 [12]; Cohort for the eight observational series; and Case-Series or Case-Report for the case-level publications. Full item-level appraisals for each study and each design are provided in Supplementary Tables S3–S6.

***Abbreviations:*** *CDC = collecting duct carcinoma; ICI = immune checkpoint inhibitor; JBI = Joanna Briggs Institute; PFS = progression-free survival; OS = overall survival; RCT = randomised controlled trial.*
