# Supplementary material for: Immunotherapy in Bellini Duct Carcinoma: A Systematic Review
Source: Oncol Res. 2026 Sep 14;34(10):2. doi: 10.32604/or.2026.081674 (PMC13591354; doi:10.32604/or.2026.081674)
Supplement: Supplementary file 1 [file OncolRes-34-81674-s001.zip › OR-81674_Supplementary_Table_S1.docx]

**Supplementary Table S1.** Individual-patient data (IPD) summary of the 16 case-level patients included in the review, and pooled descriptive statistics.

**Part A. Individual-patient data.**

| **Patient # (Study, Year)** | **Age (y)** | **Sex** | **Metastatic sites** | **Line of therapy** | **Regimen** | **Best response (RECIST)** | **PD-L1** | **Duration / Follow-up** | **Notes / AEs** |
| --- | --- | --- | --- | --- | --- | --- | --- | --- | --- |
| 1 — Mizutani, 2017 [27] | 64 | M | Lung (nodes) | Multiline | Nivolumab | CR (lung), SD (nodes) | High | NR | NR |
| 2 — Rimar, 2016 [22] | 73 | M | Lung | Multiline | Nivolumab | PR | High | NR | NR |
| 3 — Yasuoka, 2018 [28] | 53 | F | Liver, bone | Multiline | Nivolumab | PR at 3 mo | NA | 5 cycles | No AEs during 5 cycles |
| 4 — Zeng, 2020 [34] | 73 | NR | Bone | 2L | Personalised neoantigen IO | SD + clinical benefit | NA | 6 cycles | No obvious side effects |
| 5 — Pyrgidis, 2023 [33] | 71 | M | Bone, liver | 2L | Avelumab | SD | NA | NR | Fatal pneumonia on avelumab |
| 6 — Watanabe, 2019 [23] | 44 | F | Nodal | 1L | Nivolumab + ipilimumab | CR | NA | >36 mo (ongoing CR) | No irAE reported |
| 7 — Wu, 2021 [24] | 59 | F | Peritoneal, L adrenal | Multiline | Nivolumab (± prior sorafenib) | CR >6 y | NA | >72 mo (ongoing) | Prior sunitinib discontinued for proteinuria |
| 8 — Liu, 2010 [35] | 33 | M | Pleural | 1L | Cascade-primed immune cells | Symptom relief | NA | NR | NR |
| 9 — Fuu, 2022 [25] | 70 | M | Lung, liver | 1L | Nivolumab + ipilimumab | CR >3 y | >10% | >36 mo (ongoing) | NR |
| 10 — Zhou W., 2021 [32] | 67 | M | Lung, bone | 1L | Camrelizumab + pazopanib → axitinib + sintilimab | PR; lung cleared; >14 mo | Positive | >14 mo | Drug-induced hepatitis on pazopanib+camrelizumab; steroids; HTN on second regimen |
| 11 — Tamada, 2022 [30] (case 1) | 62 | M | Bone | NR | Pembrolizumab + axitinib | PR | Negative | NR | NR |
| 12 — Tamada, 2022 [30] (case 2) | 71 | M | Adrenal, lung | NR | Pembrolizumab + axitinib | PR | Negative | NR | NR |
| 13 — Abe, 2024 [26] | 67 | F | Liver, peritoneum | 2L | Pembrolizumab monotherapy | CR >5 y | NA | >60 mo (ongoing) | NR |
| 14 — Atagi, 2023 [31] | 69 | M | Adrenal | 1L | Pembrolizumab + axitinib | PR | NA | NR | NR |
| 15 — Danno, 2021 [29] (case 1) | 75 | F | Bone | 1L | Nivolumab + ipilimumab | SD | NA | NR | NR |
| 16 — Danno, 2021 [29] (case 2) | 79 | F | Lung, nodes | 1L | Nivolumab + ipilimumab | PR | NA | NR | NR |

*CR = complete response; PR = partial response; SD = stable disease; PD = progressive disease; RECIST = Response Evaluation Criteria in Solid Tumors; PD-L1 = programmed death-ligand 1; 1L/2L = first-/second-line therapy; F = female; M = male; NR = not reported; NA = not available; HTN = hypertension; IO = immunotherapy; irAE = immune-related adverse event.*

**Part B. Pooled descriptive statistics.**

| **Variable** | **n / N (%) or summary** | **Notes** |
| --- | --- | --- |
| Total patients (denominator N) | 16 / 16 (100%) | Independent case-level patients from 14 publications (overlap-verified) |
| Median age (range) | 66 y (33–79) | Age reported for 15/16 patients; one case (Zeng 2020) reported age but not sex |
| Sex (Male) | 8 / 15 (53%) | One case did not report sex; excluded from denominator |
| First-line therapy | 8 / 16 (50%) | 1L vs 2L/multiline split |
| Best response — CR | 4 / 16 (25%) | Wu, Watanabe, Fuu, Abe (durable CRs) |
| Best response — PR | 7 / 16 (44%) | Rimar, Yasuoka, Mizutani (lung-focus), Zhou W., Tamada×2, Atagi, Danno-2 |
| Best response — SD/clinical benefit | 4 / 16 (25%) | Zeng (neoantigen), Pyrgidis (avelumab), Liu (cell therapy), Danno-1 |
| Best response — PD / primary progression | 0 / 16 (0%) | No primary progression documented (selection bias likely) |
| Objective response rate (ORR = CR+PR) | 11 / 16 (69%) | High ORR reflects publication bias in case-level literature |
| Disease control rate (DCR = CR+PR+SD) | 15 / 16 (94%) | Same caveat |
| Duration of response (months, median where reported) | Not estimable | Heterogeneous follow-up; at least 9/16 had response sustained ≥12 months |
| Regimen: anti-PD-1 monotherapy | 5 / 16 (31%) | Nivolumab ×4, pembrolizumab ×1 |
| Regimen: dual checkpoint (nivolumab + ipilimumab) | 4 / 16 (25%) | Watanabe, Fuu, Danno×2 |
| Regimen: ICI + TKI (anti-PD-1/L1 + axitinib or pazopanib) | 4 / 16 (25%) | Tamada×2, Atagi, Zhou W. |
| Regimen: other (avelumab, neoantigen vaccine, cell therapy) | 3 / 16 (19%) | Pyrgidis, Zeng, Liu |
| PD-L1 positive (when reported) | 4 / 7 reported (57%) | PD-L1 status reported in only 7/16; 4 positive, 3 negative |
| Serious adverse events documented | 2 / 16 (13%) | Pyrgidis (fatal pneumonia); Zhou W. (drug-induced hepatitis, managed with steroids) |

**Interpretative notes.**

*The figures above are crude proportions derived from published case-level reports. They are presented for descriptive purposes only and must not be interpreted as population-level estimates of ICI efficacy in CDC. The most important caveats are: (i) near-certain positive-outcome selection bias (no primary progression event is documented across the 16 cases, which is implausible for a disease of this aggressiveness in an unselected population); (ii) heterogeneity in line of therapy, regimen, and follow-up; (iii) variable reporting of PD-L1 status, with status reported in only 7/16 patients; (iv) overlap with larger observational cohorts in the review was verified and excluded. Formal Kaplan–Meier aggregation and forest plots were not constructed because line-of-therapy, regimen class, and censoring definitions differed too markedly across case-level reports to support valid aggregation. Any pooled effect-size estimate should therefore be regarded as hypothesis-generating.*
