# Supplementary material for: Immunotherapy in Bellini Duct Carcinoma: A Systematic Review
Source: Oncol Res. 2026 Sep 14;34(10):2. doi: 10.32604/or.2026.081674 (PMC13591354; doi:10.32604/or.2026.081674)
Supplement: Supplementary file 1 [file OncolRes-34-81674-s001.zip › OR-81674_Supplementary_Table_S2.docx]

**Supplementary Table S2.** Durable complete responses (CR) of ≥ 3 years in CDC patients treated with immunotherapy-based regimens, as identified in this systematic review.

| **Study (Author, Year) [Ref]** | **Age / Sex** | **Metastatic sites** | **Line of therapy** | **Regimen** | **PD-L1 status** | **Duration of CR (months)** | **Notes** |
| --- | --- | --- | --- | --- | --- | --- | --- |
| Wu, 2021 [24] | 59 / F | Peritoneal, left adrenal | Multiline (after prior sunitinib) | Nivolumab (± prior sorafenib) | NR | >72 mo (>6 y) | Ongoing CR; switch due to prior sunitinib-related proteinuria |
| Abe, 2024 [26] | 67 / F | Liver, peritoneum | Second-line | Pembrolizumab monotherapy | NR | >60 mo (>5 y) | CR maintained on ongoing pembrolizumab |
| Fuu, 2022 [25] | 70 / M | Lung, liver | First-line | Nivolumab + ipilimumab | >10% | >36 mo (>3 y) | PD-L1 high; no AE reported |
| Watanabe, 2019 [23] | 44 / F | Nodal | First-line | Nivolumab + ipilimumab | NR | >36 mo (CR duration approx.) | First CDC CR reported with dual ICI in first line |
| Zeng, 2025 (case A) [16] | NR | NR | Multiline | ICI-based combination (see Wang 2025) | NR | >56 mo | Sustained CR in ICI-based regimen |
| Zeng, 2025 (case B) [16] | NR | NR | Multiline | ICI-based combination (see Wang 2025) | NR | >64 mo | Sustained CR in ICI-based regimen |
| Hajmusa, 2025 (context) | NR / F | Para-aortic node recurrence | Multiline after systemic progression | Nivolumab + SBRT (20–25 Gy/5 fx) | NR; CDKN2A homozygous deletion | Ongoing metabolic CR (Jan 2025) | Case reported post-search (Oct 2025); included here as contextual supporting evidence |

*CR = complete response; CDC = collecting duct carcinoma; F = female; M = male; ICI = immune checkpoint inhibitor; NR = not reported; SBRT = stereotactic body radiotherapy; y = years; mo = months.*

*Note: duration of CR corresponds to the reported follow-up during which CR was maintained at the time of the latest publication; ongoing CRs may be longer than stated. The Hajmusa 2025 case is included as contextual evidence although it was published after our updated literature search (11 April 2026) and therefore was not formally included in the systematic synthesis; it is listed here for readers’ convenience given its direct relevance to the durable-response phenotype in CDC.*
