# Supplementary material for: Immunotherapy in Bellini Duct Carcinoma: A Systematic Review
Source: Oncol Res. 2026 Sep 14;34(10):2. doi: 10.32604/or.2026.081674 (PMC13591354; doi:10.32604/or.2026.081674)
Supplement: Supplementary file 1 [file OncolRes-34-81674-s001.zip › OR-81674_Supplementary_Table_S3-S6.docx]

**SUPPLEMENT**

The supplementary tables have been reorganised so that each table now follows the standard sequence (title, table body, and footnote with item definitions where applicable). Tables have also been renumbered consecutively (S1–S6) to avoid the previous panel-letter labelling (S1a–S1d), as requested.

Below is the item‑level risk‑of‑bias (RoB) matrix for all included studies, using the Joanna Briggs Institute (JBI) checklists by design. Ratings: Y = Yes; N = No; U = Unclear; NA = Not applicable. Item wording is reproduced/condensed from the relevant JBI tools. Item‑level judgments are based on the full texts (e.g., Yasuoka 2018; Zhou 2021; Zeng 2020; Li Zhou 2022).

## Supplementary Table S3. JBI Quasi‑Experimental (single‑arm prospective) — item‑level RoB.

| **Study (Author/Year [Ref])** | **Q1** | **Q2** | **Q3** | **Q4** | **Q5** | **Q6** | **Q7** | **Q8** | **Q9** |
| --- | --- | --- | --- | --- | --- | --- | --- | --- | --- |
| Vogelzang, 2020 (CheckMate 374) [12] | Y | Y | Y | N | Y | U | Y | Y | Y |

## Table footnote — Items (JBI Quasi‑Experimental; 9): Q1 cause/effect clear · Q2 participants in comparisons similar · Q3 participants treated similarly (apart from exposure) · Q4 control group present · Q5 multiple pre/post measurements · Q6 follow‑up complete or differences described · Q7 outcomes measured the same way · Q8 outcomes measured reliably · Q9 appropriate analysis. Y = Yes; N = No; U = Unclear.

**Supplementary Table S4. JBI Cohort — item‑level RoB.**

| **Study (Author/Year [Ref])** | **C1** | **C2** | **C3** | **C4** | **C5** | **C6** | **C7** | **C8** | **C9** | **C10** | **C11** |
| --- | --- | --- | --- | --- | --- | --- | --- | --- | --- | --- | --- |
| Guillaume, 2022 [14] | NA | NA | Y | U | U | Y | Y | U | U | U | Y |
| Zhou (Li), 2022 [15] | NA | NA | Y | Y | Y | Y | Y | Y | U | U | Y |
| Buti, 2023 [17] | NA | NA | Y | U | U | Y | Y | U | U | U | Y |
| Izumi, 2023 [18] | NA | NA | Y | U | U | Y | Y | U | U | U | Y |
| Izarn, 2023 [19] | NA | NA | Y | U | U | Y | U | U | U | U | U |
| Kwon, 2014 [20] | NA | NA | Y | U | U | Y | U | U | U | U | U |
| Chen, 2022 [21] | NA | NA | Y | U | U | Y | U | U | U | U | U |

## Table footnote — Items (JBI Cohort; 11): C1 groups similar & recruited similarly · C2 exposure measured similarly in groups · C3 exposure measured validly/reliably · C4 confounders identified · C5 strategies to deal with confounders stated · C6 outcome‑free at start · C7 outcomes measured validly/reliably · C8 follow‑up time sufficient · C9 follow‑up complete or differences described · C10 strategies for incomplete follow‑up · C11 appropriate analysis. Y = Yes; U = Unclear; NA = Not applicable.

**Supplementary Table S5. JBI Case‑Series — item‑level RoB.**

| **Study (Author/Year [Ref])** | **S1** | **S2** | **S3** | **S4** | **S5** | **S6** | **S7** | **S8** | **S9** | **S10** |
| --- | --- | --- | --- | --- | --- | --- | --- | --- | --- | --- |
| Danno, 2021 [29] | U | U | Y | U | U | Y | Y | Y | U | NA |
| Tamada, 2022 [30] | U | U | Y | U | U | Y | Y | Y | U | NA |

## Table footnote — Items (JBI Case‑Series; 10): S1 clear inclusion criteria · S2 condition measured in a standard, reliable way for all · S3 valid methods for case identification · S4 consecutive inclusion · S5 complete inclusion · S6 demographics reported · S7 clinical info reported · S8 outcomes/ follow‑up reported · S9 site(s) demographics reported · S10 appropriate statistical analysis. Note: many retrospective series are single‑group by design; JBI group‑comparison items (C1–C2) are marked NA. Y = Yes; U = Unclear; NA = Not applicable.

## Supplementary Table S6. JBI Case‑Report — item‑level RoB.

| **Study (Author/Year [Ref])** | **R1** | **R2** | **R3** | **R4** | **R5** | **R6** | **R7** | **R8** |
| --- | --- | --- | --- | --- | --- | --- | --- | --- |
| Rimar, 2016 [22] | Y | Y | Y | Y | Y | Y | U | Y |
| Watanabe, 2019 [23] | Y | Y | Y | Y | Y | Y | U | Y |
| Wu, 2021 [24] | Y | U | Y | Y | Y | Y | Y | Y |
| Fuu, 2022 [25] | Y | U | Y | Y | Y | Y | U | Y |
| Abe, 2024 [26] | Y | Y | Y | Y | Y | Y | U | Y |
| Mizutani, 2017 [27] | Y | U | Y | Y | Y | Y | U | Y |
| Yasuoka, 2018 [28] | Y | Y | Y | Y | Y | Y | U | Y |
| Atagi, 2023 [31] | Y | U | Y | Y | Y | Y | U | Y |
| Zhou W., 2021 [32] | Y | Y | Y | Y | Y | Y | Y | Y |
| Pyrgidis, 2023 [33] | Y | U | Y | Y | Y | Y | Y | Y |
| Zeng, 2020 [34] | Y | Y | Y | Y | Y | Y | Y | Y |
| Liu, 2010 [35] | Y | U | Y | Y | Y | Y | U | Y |

## Table footnote — Items (JBI Case‑Report; 8): R1 patient demographics described · R2 history presented (timeline) · R3 current clinical condition described · R4 diagnostic tests/assessment methods & results described · R5 intervention(s) clearly described · R6 post‑intervention clinical condition described · R7 adverse events/side‑effects identified · R8 takeaway lessons provided Y = Yes; U = Unclear.

### AE = adverse event; CDC = collecting duct carcinoma; ICI = immune checkpoint inhibitor; JBI = Joanna Briggs Institute; nccRCC = non‑clear‑cell renal cell carcinoma; PFS = progression‑free survival.
