# Supplementary material for: Immunotherapy in Bellini Duct Carcinoma: A Systematic Review
Source: Oncol Res. 2026 Sep 14;34(10):2. doi: 10.32604/or.2026.081674 (PMC13591354; doi:10.32604/or.2026.081674)
Supplement: Supplementary file 1 [file OncolRes-34-81674-s001.zip › Supplementary_Material_S1.docx]

| **Section and Topic** | **Item No** | **Checklist item** | **Reported on Page Number/Line Number** | **Reported on Section/Paragraph** |
| --- | --- | --- | --- | --- |
| **TITLE** | | |  |  |
| Title | 1 | Identify the report as a systematic review. | Title page | Title: "Immunotherapy in Bellini Duct Carcinoma: A Systematic Review" — explicitly identifies the report as a systematic review. |
| **ABSTRACT** | | |  |  |
| Abstract | 2 | See the PRISMA 2020 for Abstracts checklist. | Abstract page | Abstract: structured abstract follows the PRISMA 2020 for Abstracts checklist; covers background, methods, results, discussion, funding and registration (PROSPERO CRD420251022132). |
| **INTRODUCTION** | | |  |  |
| Rationale | 3 | Describe the rationale for the review in the context of existing knowledge. | Introduction, paragraph 1–4 | Introduction: rationale provided in the context of CDC rarity, aggressiveness, limited efficacy of standard therapies, and emerging ICI evidence in non-clear-cell RCC. |
| Objectives | 4 | Provide an explicit statement of the objective(s) or question(s) the review addresses. | Introduction, last paragraph | Introduction: explicit statement of the objective — "to consolidate efficacy and safety data for immunotherapy in Bellini duct carcinoma". |
| **METHODS** | | |  |  |
| Eligibility criteria | 5 | Specify the inclusion and exclusion criteria for the review and how studies were grouped for the syntheses. | Methods — Eligibility criteria | Methods/Eligibility criteria: PICOS framework specified; inclusion/exclusion criteria stated; study groupings for synthesis (prospective / observational / case-level) defined. |
| Information sources | 6 | Specify all databases, registers, websites, organisations, reference lists and other sources searched or consulted to identify studies. Specify the date when each source was last searched or consulted. | Methods — Search strategy | Methods/Search strategy: two electronic databases searched (PubMed, Web of Science); search date 29 March 2025. Embase not formally searched, declared as a limitation. Targeted post-search monitoring of key genitourinary-oncology journals and of ClinicalTrials.gov performed on 11 April 2026 to capture studies appearing between original search and submission; records thus identified are presented as a distinct pathway in the PRISMA flow diagram under "other methods". |
| Search strategy | 7 | Present the full search strategies for all databases, registers and websites, including any filters and limits used. | Methods — Search strategy | Methods/Search strategy: full PubMed query provided verbatim; adapted for Web of Science. No formal Embase search performed. Targeted monitoring used structured journal browsing (Annals of Oncology, European Urology, Journal of Clinical Oncology, Clinical Genitourinary Cancer, Journal for ImmunoTherapy of Cancer) and ClinicalTrials.gov search with CDC-specific terms. English-language restriction acknowledged as a limitation. |
| Selection process | 8 | Specify the methods used to decide whether a study met the inclusion criteria of the review, including how many reviewers screened each record and each report retrieved, whether they worked independently, and if applicable, details of automation tools used in the process. | Methods — Study selection | Methods/Study Selection: two-step screening (title/abstract then full text) by four independent reviewers (ADLS, JDBF, SW, MNR); disagreements resolved by two additional reviewers (BFM, ABA); PRISMA flow diagram used. "Other methods" identification pathway comprises 5 records from citation searching and 2 records from targeted post-search monitoring (SUNNIFORECAST; Zeng et al. 2025), total 7 records, all assessed at full-text level. |
| Data collection process | 9 | Specify the methods used to collect data from reports, including how many reviewers collected data from each report, whether they worked independently, any processes for obtaining or confirming data from study investigators, and if applicable, details of automation tools used in the process. | Methods — Data extraction | Methods/Data Extraction: four reviewers independently extracted data; cross-checking by reviewer pairs; dedicated overlap-verification procedure confirming the 16 case-level patients are independent individuals. |
| Data items | 10a | List and define all outcomes for which data were sought. Specify whether all results that were compatible with each outcome domain in each study were sought (e.g. for all measures, time points, analyses), and if not, the methods used to decide which results to collect. | Methods — Data items | Methods/Data items: outcomes defined a priori — ORR, DCR, PFS, OS, and safety (adverse events). All compatible results sought for each outcome. |
|  | 10b | List and define all other variables for which data were sought (e.g. participant and intervention characteristics, funding sources). Describe any assumptions made about any missing or unclear information. | Methods — Data items | Methods/Data items: additional variables collected — study design, sample size (CDC-specific and total), demographics, regimen, line of therapy, PD-L1 status, molecular biomarkers where reported, funding source. Assumptions about missing data explicitly stated in individual study rows of Tables 1–3. |
| Study risk of bias assessment | 11 | Specify the methods used to assess risk of bias in the included studies, including details of the tool(s) used, how many reviewers assessed each study and whether they worked independently, and if applicable, details of automation tools used in the process. | Methods — Risk of bias assessment | Methods/Risk of Bias: JBI critical appraisal tools applied (cohort, case-series, case-report tools as appropriate); rationale for not using RoB 2 / ROBINS-I explained; immortal time bias explicitly considered for observational cohorts (references 10, 11); four reviewers (ADLS, JDBF, SW, MNR) independently appraised each study, with disagreements resolved by consensus involving two senior reviewers (BFM, ABA). |
| Effect measures | 12 | Specify for each outcome the effect measure(s) (e.g. risk ratio, mean difference) used in the synthesis or presentation of results. | Methods — Data Synthesis | Methods/Data Synthesis: effect measures specified — ORR as proportion (n/N); DCR as proportion; OS and PFS as medians in months (95% CI where reported). No pooled effect measures computed. |
| Synthesis methods | 13a | Describe the processes used to decide which studies were eligible for each synthesis (e.g. tabulating the study intervention characteristics and comparing against the planned groups for each synthesis (item #5)). | Methods — Data Synthesis | Methods/Data Synthesis: synthesis groupings (prospective interventional, observational cohorts, case-level reports) determined a priori based on study design; tabulation of intervention characteristics used to map studies to syntheses. |
|  | 13b | Describe any methods required to prepare the data for presentation or synthesis, such as handling of missing summary statistics, or data conversions. | Methods — Data Synthesis | Methods/Data Synthesis: data preparation — harmonisation of response definitions (RECIST v1.0, v1.1, investigator-assessed); conversion of follow-up times to months; explicit handling of unreported subgroup statistics. |
|  | 13c | Describe any methods used to tabulate or visually display results of individual studies and syntheses. | Methods — Data Synthesis | Methods/Data Synthesis: results tabulated per study design block (Tables 1–3) with uniform column structure; supplementary traffic-light summary for RoB (Supplementary Figure S1). |
|  | 13d | Describe any methods used to synthesize results and provide a rationale for the choice(s). If meta-analysis was performed, describe the model(s), method(s) to identify the presence and extent of statistical heterogeneity, and software package(s) used. | Methods — Data Synthesis | Methods/Data Synthesis: a formal meta-analysis was not conducted; three explicit sources of heterogeneity enumerated (study design, intervention, outcome definition); I² not computed, with justification. Descriptive pooled ORR/DCR estimates provided as hypothesis-generating only. |
|  | 13e | Describe any methods used to explore possible causes of heterogeneity among study results (e.g. subgroup analysis, meta-regression). | Methods — Data Synthesis | Methods/Data Synthesis: possible causes of heterogeneity addressed qualitatively (line of therapy, regimen type, PD-L1 status where reported); no formal subgroup meta-regression performed because of insufficient quantitative data. |
|  | 13f | Describe any sensitivity analyses conducted to assess robustness of the synthesized results. | Not applicable | Not applicable — no formal synthesis with pooled effect estimates was performed, therefore sensitivity analyses of pooled effect estimates were not conducted. |
| Reporting bias assessment | 14 | Describe any methods used to assess risk of bias due to missing results in a synthesis (arising from reporting biases). | Methods — Risk of Bias / Discussion | Methods/Risk of Bias + Discussion/Limitations: reporting-bias risk addressed qualitatively, focusing on publication bias in case-level literature (near-certain positive-outcome selection) and language-restriction bias. Formal Egger/funnel-plot assessment not feasible due to heterogeneity; this is explicitly acknowledged. |
| Certainty assessment | 15 | Describe any methods used to assess certainty (or confidence) in the body of evidence for an outcome. | Methods — Risk of Bias | Methods/Risk of Bias: overall certainty of evidence discussed qualitatively per study design block; GRADE not formally applied because of inherent heterogeneity and the predominance of case-level evidence; this decision is justified. |
| **RESULTS** | | |  |  |
| Study selection | 16a | Describe the results of the search and selection process, from the number of records identified in the search to the number of studies included in the review, ideally using a flow diagram. | Results — Study selection | Results/Study Selection: numbers of records identified, screened, assessed for eligibility, and included reported; PRISMA flow diagram (Figure 1) provided, including the citation-search pathway (5 additional records). |
|  | 16b | Cite studies that might appear to meet the inclusion criteria, but which were excluded, and explain why they were excluded. | Results — Study selection | Results/Study Selection: reasons for exclusion at full-text stage documented in the PRISMA flow diagram and corresponding text; a supplementary list of excluded studies with reasons is available on request. |
| Study characteristics | 17 | Cite each included study and present its characteristics. | Results — Table 1, 2, 3 | Results: each included study cited and characterised in Tables 1 (prospective), 2 (observational), and 3 (case-level). Supplementary Table S1 provides full IPD for case-level patients. |
| Risk of bias in studies | 18 | Present assessments of risk of bias for each included study. | Results — RoB + Table 4 | Results/Risk of Bias + Table 4: RoB judgements presented per study with per-study "Key concerns" rationale; Supplementary Tables S3–S6 give item-level JBI appraisals (one supplementary table per study design); Supplementary Figure S1 provides the graphical traffic-light summary. |
| Results of individual studies | 19 | For all outcomes, present, for each study: (a) summary statistics for each group (where appropriate) and (b) an effect estimate and its precision (e.g. confidence/credible interval), ideally using structured tables or plots. | Results — Tables 1, 2, 3 | Results: summary statistics and where reported effect measures with precision presented per study in Tables 1–3; individual patient data presented in Supplementary Table S1. |
| Results of syntheses | 20a | For each synthesis, briefly summarise the characteristics and risk of bias among contributing studies. | Results — per-block Summary paragraphs | Results: each synthesis block opens with a "Summary:" lead paragraph that briefly characterises contributing studies and their risk of bias (prospective, observational, case-level). |
|  | 20b | Present results of all statistical syntheses conducted. If meta-analysis was done, present for each the summary estimate and its precision (e.g. confidence/credible interval) and measures of statistical heterogeneity. If comparing groups, describe the direction of the effect. | Results — Pooled descriptive analysis | Results: crude pooled ORR/DCR presented for the case-level IPD in Supplementary Table S1; explicit caveats that this is descriptive and hypothesis-generating only; no confidence intervals or I² reported (by design). |
|  | 20c | Present results of all investigations of possible causes of heterogeneity among study results. | Not applicable | Not applicable — no formal heterogeneity investigation performed because no meta-analytic synthesis was undertaken. |
|  | 20d | Present results of all sensitivity analyses conducted to assess the robustness of the synthesized results. | Not applicable | Not applicable — no sensitivity analyses of pooled estimates performed. |
| Reporting biases | 21 | Present assessments of risk of bias due to missing results (arising from reporting biases) for each synthesis assessed. | Discussion — Limitations | Discussion/Limitations of the evidence: reporting-bias assessment discussed qualitatively; publication bias in case-level literature acknowledged openly with quantitative caveat that real-world ORR is likely closer to the lower bound (~20%) of the crude pooled estimate. |
| Certainty of evidence | 22 | Present assessments of certainty (or confidence) in the body of evidence for each outcome assessed. | Discussion — Limitations | Discussion/Limitations of the evidence: overall certainty explicitly framed as low, given predominance of retrospective / case-level evidence, tiny CDC subsets in prospective trials, and heterogeneity of endpoints. |
| **DISCUSSION** | | |  |  |
| Discussion | 23a | Provide a general interpretation of the results in the context of other evidence. | Discussion — Efficacy synthesis | Discussion/Efficacy synthesis: general interpretation provided in context of other RCC evidence (clear-cell ICI efficacy; nccRCC evidence base; historical gemcitabine–platinum ORR). |
|  | 23b | Discuss any limitations of the evidence included in the review. | Discussion — Limitations of the evidence | Discussion/Limitations of the evidence: limitations of the evidence (design, sample size, outcome heterogeneity, language restriction, publication bias) discussed explicitly. |
|  | 23c | Discuss any limitations of the review processes used. | Discussion — Limitations of the review | Discussion/Limitations of the review: limitations of the review process discussed — databases included (and excluded), language restriction, absence of formal meta-analytic pooling, no GRADE. |
|  | 23d | Discuss implications of the results for practice, policy, and future research. | Discussion — Future directions | Discussion/Future directions: implications for practice (promising but provisional signals; selection of patients for ICI-based regimens); for policy (rare-disease research infrastructure, basket trials, federated registries); and for future research (CDC-dedicated biomarker-stratified trials, adaptive designs). |
| **OTHER INFORMATION** | | |  |  |
| Registration and protocol | 24a | Provide registration information for the review, including register name and registration number, or state that the review was not registered. | Methods — Methodology, first paragraph | Methods: review prospectively registered in PROSPERO — Registration ID CRD420251022132. |
|  | 24b | Indicate where the review protocol can be accessed, or state that a protocol was not prepared. | Methods — Methodology, first paragraph | Methods: protocol is accessible via the PROSPERO registry using the ID CRD420251022132. |
|  | 24c | Describe and explain any amendments to information provided at registration or in the protocol. | Methods — Search strategy / Study selection | Amendments to the protocol: search update on 11 April 2026 to capture studies published between the original search (29 March 2025) and manuscript submission, by extending the PubMed and Web of Science searches to that date and by adding targeted post-search monitoring of high-impact genitourinary-oncology journals and of relevant ClinicalTrials.gov records, in response to peer-review recommendations; documented in the Methods — Search strategy section with the updated screening results. |
| Support | 25 | Describe sources of financial or non-financial support for the review, and the role of the funders or sponsors in the review. | End-matter — Funding Statement | Funding Statement: provided in end-matter; sources of financial and non-financial support described; role of funders/sponsors (if any) explicitly stated. |
| Competing interests | 26 | Declare any competing interests of review authors. | End-matter — Conflict of Interest | Conflict of Interest: provided in end-matter; authors declare any competing interests or absence thereof. |
| Availability of data, code and other materials | 27 | Report which of the following are publicly available and where they can be found: template data collection forms; data extracted from included studies; data used for all analyses; analytic code; any other materials used in the review. | End-matter — Availability of Data | Availability of Data and Materials: provided in end-matter; the review synthesises data from previously published studies; data extracted from each study are reported in Tables 1–3 and Supplementary Tables S1–S6; the data-extraction form and full item-level JBI appraisals are available from the corresponding author upon reasonable request. |

*As the checklist was provided upon initial submission, the page number/line number reported may be changed due to copyediting and may not be referable in the published version. In this case, the section/paragraph may be used as an alternative reference.
